# Comparison of RT-dPCR and RT-qPCR and the effects of freeze–thaw cycle and glycine release buffer for wastewater SARS-CoV-2 analysis

**DOI:** 10.1038/s41598-022-25187-1

**Published:** 2022-11-30

**Authors:** Bonnie Jaskowski Huge, Devin North, C. Bruce Mousseau, Kyle Bibby, Norman J. Dovichi, Matthew M. Champion

**Affiliations:** 1 Department of Chemistry and Biochemistry, University of Notre Dame, Notre Dame, IN 46556 USA; 2 Department of Civil and Environmental Engineering and Earth Sciences, University of Notre Dame, Notre Dame, IN 46556 USA; 3 Berthiaume Institute for Precision Health, University of Notre Dame, Notre Dame, IN 46556 USA

**Keywords:** Microbiology, Analytical chemistry

## Abstract

Public health efforts to control the severe acute respiratory syndrome coronavirus 2 (SARS-CoV-2) pandemic rely on accurate information on the spread of the disease in the community. Acute and surveillance testing has been primarily used to characterize the extent of the disease. However, obtaining a representative sample of the human population is challenging because of limited testing capacity and incomplete testing compliance. Wastewater-based epidemiology is an agnostic alternative to surveillance testing that provides an average sample from the population served by the treatment facility. We compare the performance of reverse transcription quantitative PCR (RT-qPCR) and reverse transcription digital droplet PCR (RT-dPCR) for analysis of SARS-CoV-2 RNA in a regional wastewater treatment facility in northern Indiana, USA from the earliest stages of the pandemic. 1-L grab samples of wastewater were clarified and concentrated. Nucleic acids were extracted from aliquots and analyzed in parallel using the two methods. Synthetic viral nucleic acids were used for method development and generation of add-in standard-curves. Both methods were highly sensitive in detecting SARS-CoV-2 in wastewater, with detection limits as low as 1 copy per 500 mL wastewater. RT-qPCR and RT-dPCR provided essentially identical coefficients of variation (s/$\overline{\mathrm{x} }$ = 0.15) for triplicate measurements made on wastewater samples taken on 16 days. We also observed a sevenfold decrease in viral load from a grab sample that was frozen at – 80 °C for 92 days compared to results obtained without freezing. Freezing samples before analysis should be discouraged. Finally, we found that treatment with a glycine release buffer resulted in a fourfold inhibition in RT-qPCR signal; treatment with a glycine release buffer also should be discouraged. Despite their prevalence and convenience in wastewater analysis, glycine release and freezing samples severely and additively (~ tenfold) degraded recovery and detection of SARS-CoV-2.

## Introduction

The ongoing coronavirus disease (COVID-19) pandemic, caused by the severe acute respiratory syndrome coronavirus 2 (SARS-CoV-2), is a global public health emergency^1^. SARS-CoV-2 is an enveloped, single-stranded RNA virus in the *Orthocoronavirinae* subfamily, categorized by high infectivity rates and a high proportion of asymptomatic infections^2^.

Public health actions require accurate estimates of COVID-19 in the community. Obtaining a representative sample^3^ from a human population is challenging because of limited testing capacity and incomplete compliance.

SARS-CoV-2 enters cells via angiotensin converting enzyme II (ACE2) receptors. Infection causes respiratory symptoms in most infections, but also leads to gastrointestinal infection, with subsequent presence of the virus in feces^4–6^. In principle, municipal wastewater analysis provides an aggregate viral sample of the community that it serves, and SARS-CoV-2 levels in wastewater can act as a proxy for the prevalence of SARS-CoV-2 in the community^7–11^.

Analysis of viral loads in wastewater is challenging. The chemical and biological properties of wastewater vary based on the characteristics of the sewershed, such as residential and industrial contribution, along with weather, seasonal, and temporal flow variations. Several reports characterize SARS-CoV-2 concentrations from composite and ‘grab’ samples over 24-h study windows^11–17^.

Viruses in wastewater are highly dilute compared to clinical specimens and viral load can be below the limit of detection for quantitative PCR based approaches; thus, sample concentration is usually necessary to enhance detection. Confounding analysis, SARS-CoV-2 is present in a sea of other human, animal, and bacterial viruses in an organic rich matrix^16–18^.

Numerous concentration methods have been applied to improve SARS-CoV-2 detection from wastewater samples, but heterogeneity among wastewater samples, even from the same wastewater treatment plant, makes it difficult to create and compare universally optimized methods^18–21^. Concentration of viruses also co-concentrates PCR inhibitors within wastewater, introducing biases from the concentration method itself in downstream molecular applications^22,23^. These inhibitors include a range of organic compounds and salts.

Reverse transcription quantitative PCR (RT-qPCR) and reverse transcription digital droplet PCR (RT-dPCR) are the most popular methods for virus quantitation. In both cases, reverse transcriptase converts the RNA virus into a DNA strand that can be amplified by PCR. RT-qPCR measures the number of cycles in a polymerase chain reaction to generate a signal that exceeds a threshold, the Ct value. The Ct value is inversely exponentially related to the concentration of virus in the sample, dilute samples generate relatively large Ct values, and calibration is required to quantitate the virus concentration. In contrast, RT-dPCR disperses a sample in a large number of minute droplets. The dilute virus sample is distributed according to the Poisson distribution among the drops. After PCR amplification, the fraction of drops showing a positive signal is converted to the virus concentration in the sample based on the Poisson distribution.

RT-qPCR is commonly used because of its modest entry costs and the wide availability of consumables. However, RT-qPCR is an empirical technique, relying on standard curves for quantification, whereas RT-dPCR does not need calibration. Several studies have shown that RT-dPCR is more sensitive than RT-qPCR for target quantification, especially for environmental samples with matrix-associated inhibitors. Rački et al. characterized the detection and quantitation of pepper mild mottle virus (PMMoV) from a variety of environmental samples, including wastewater, as well as in the presence of isolated inhibitors using both RT-dPCR and RT-qPCR^24^. The binary positive/negative quantification method of RT-dPCR makes it less susceptible to probe inhibition and less sensitive to reductions in exponential amplification efficiency. Tozaki et al*.* compared RT-qPCR and RT-dPCR for gene-doping control^25^. They demonstrated that RT-dPCR resulted in more consistent quantitation without complete match of the primers with the target sequence, while Ct increased for RT-qPCR with mismatched primers.

The Center for Disease Control in the United States developed N1 and N2 RT-qPCR assays that target gene regions within SARS-CoV-2^26^. After initial missteps^27^, these assays were rapidly adopted for population screening^28^.

Here we present a study of SARS-CoV-2 RNA in wastewater during the onset of COVID-19 cases in northern Indiana, USA. Aliquots of purified viral nucleic acids were prepared for detection and quantification in parallel by RT-qPCR and RT-dPCR. The use of a glycine release buffer and storage of samples at − 80 °C before analysis were also investigated.

## Experimental section

### Materials and methods

All reagents were analytical grade and purchased from Sigma-Aldrich (St. Louis, MO USA) unless otherwise noted. Solutions were prepared from deionized-distilled water (ddH_2_O) obtained from a Barnstead Nanopure System (Thermo-Fisher Scientific). Consumables and glassware were purchased sterile or autoclaved prior to use.

### Wastewater sampling

Wastewater sampling was conducted at a regional wastewater treatment plant (WWTP) located in St. Joseph County in northern Indiana, USA. The WWTP serves 56,227 residents and had an average influent flow rate of 14.09 MGD in 2020.

A total of 53 wastewater samples was collected throughout the initial outbreak of local COVID-19 cases, spanning March 12–July 24, 2020. Each sample consisted of a 1 L grab sample of primary influent or primary effluent. The number of positive cases within the surrounding county rose from zero to 3000 over the period of this analysis.

### Sample processing for virion isolation

Several sample treatment protocols were employed during this early stage of the pandemic. One set of samples was processed shortly after collection. Another set was frozen before analysis due to changes in sample handling requirements by the CDC. Another set of samples was treated with a glycine release buffer.

#### Untreated wastewater samples

Raw wastewater samples were divided into sterile 50 mL Corning Falcon™ tubes (Sigma-Aldrich) and subjected to low spin clarification (650×*g*, 4 min, 4 °C). The samples were then decanted onto a 0.2 μm PES membrane contained in a Nalgene Nunc bottle top filter unit (Sigma-Aldrich) and filtered under vacuum. The filtrate was processed as described in the virion concentration section, below.

#### Glycine-release treated wastewater samples

500 mL of raw wastewater was mixed with 50 mL of glycine release buffer (0.05 M glycine, 3% beef extract, pH 9.6) and stored at 4 °C for 20 min^29^. Glycine-release treated samples were divided into sterile 50 mL Falcon tubes and solids were pelleted by centrifugation (5000×*g*, 12 min, 4 °C). The supernatant was decanted onto a 0.2 μm PES membrane contained in a bottle top filter unit and filtered under vacuum. The filtrate was processed as described in the virion concentration section, below.

#### Subset processing

Six untreated samples were processed immediately after collection from an initial volume of approximately 1 L each. An additional aliquot of 500 mL was collected with sample 6; the raw sample was stored at − 80 °C, thawed after 92 days in storage, and processed as untreated for comparison to the aliquot that was processed without freeze/thaw (Fig. 2A).

Forty-seven samples were split into two aliquots of approximately 500 mL prior to processing. For eleven samples, one aliquot was processed immediately without treatment. For thirty-seven samples, one aliquot was treated with glycine release buffer. One aliquot was stored at − 80 °C in raw form, with one exception: Sample 43 was split into two equal aliquots, which were processed in parallel as untreated and with glycine release (Fig. 2B).

Processing of eleven of the glycine-release treated samples was limited to pre-concentration steps due to changes in CDC recommendations specific to nonclinical laboratories and concentration of wastewater samples (cdc.gov/coronavirus/2019-ncov/lab/lab-biosafety-guidelines.html#environmental).

Filtered samples were stored at − 80 °C for 22–29 days, thawed, and concentrated as described in the virion concentration section.

### Virion concentration

Samples were then transferred to Amicon-Ultra 15 Centrifugal Filter Devices with 10,000 MWCO for virion concentration. Using a swinging bucket rotor, a 15 mL aliquot of processed wastewater was loaded onto the filter device and spun at 4000×*g* for 30 min at room temperature, discarding flow through. This process was repeated until the entire wastewater sample was loaded and concentrated on the filter device. The concentrated solute was washed with sterile PBS, centrifuged, and the flow through was discarded. The washed concentrate was collected and stored at − 80 °C. The final volume was ≤ 1 mL from 500 mL raw wastewater.

### RNA extraction

The PureLink Viral RNA/DNA mini kit (Invitrogen, Carlsbad, CA, USA) was used to extract viral nucleic acids per manufacturer’s instructions. To prepare lysates, 50 μL Proteinase K, ~ 1 mL concentrated sample, and 400 μL Lysis Buffer were added to a sterile microcentrifuge tube, mixed by vortexing for 15 s, and incubated at 56 °C for 15 min. Ethanol was added to the lysate tube (final ethanol concentration of 37%), mixed by vortexing for 15 s, and the final solution was transferred to the kit’s viral spin column, where nucleic acids were bound and washed. Purified viral nucleic acids were eluted in sterile RNase-free water at final volume of approximately 50 μL. Aliquots of 6.6 μL were prepared and stored at − 80 °C to limit the number of freeze/thaw cycles prior to PCR analyses.

#### Extraction control

Heat-inactivated SARS-CoV-2 virions (ATCC, MD) were prepared for viral nucleic acid extraction at a final concentration of 0.01 ng/µL. Purified nucleic acids were eluted in sterile RNase-free water at final volume of 50 μL. Aliquots of 5 μL were stored at − 80 °C to limit freeze/thaw cycles prior to PCR analyses.

### PCR detection

SARS-CoV-2 RNA was detected and quantified using the CDC N1 assay targeting the nucleocapsid gene^28^. The N1 copy number in each sample was measured in triplicate PCR reactions using the premixed primers and probe from the 2019-nCoV RUO (IDT, Coralville, IA) for both RT-qPCR and RT-dPCR.

#### RT-qPCR

RNA in sample extracts was detected and quantified by RT-qPCR performed on the BioRad CFX96 Touch Real-Time PCR Detection system with thermal cycling performed on the C1000 Touch Thermal Cycler (BioRad, Hercules, CA). Reverse transcription of RNA and PCR amplification was performed in a single reaction using TaqPath 1-Step RT-qPCR Master Mix, CG (Thermo Fisher) per the CDC’s recommended protocol (25 °C for 2 min; 50 °C for 15 min; 95 °C for 2 min; 45 Cycles: 95 °C for 3 s, 55 °C for 30 s). Each reaction was prepared as a 20 µL volume consisting of 1× reaction mix, pre-mixed N1 primers/probe solution (500 nM forward primer, 500 nM reverse primer, 125 nM probe), and template (2 µL of nucleic acid extract). Each RT-qPCR experiment included a standard curve, positive controls, negative controls, and no-template controls (nuclease-free water).

A standard curve was generated using Genomic RNA from Severe acute respiratory syndrome-related coronavirus 2 (ATCC, VR-1986D) at final concentrations of 10,000, 1000, 100, 10, and 1 genome copies (GC)/reaction. Material extracted from heat-inactivated SARS-CoV-2 (500 GC/reaction; ATCC, VR-1986HK) served as positive control. SARS-Cov Control (IDT, 10006624) served as negative control at a final concentration of 10,000 GC/reaction.

#### RT-dPCR

RNA in sample extracts was detected and quantified by RT-dPCR performed on the BioRad QX200 Droplet Digital PCR System with thermal cycling performed on the C1000 Touch Thermal Cycler (BioRad). Reverse transcription of RNA and PCR amplification were performed in a single reaction using the One-Step RT-dPCR Advanced Kit for Probes (BioRad) per the manufacturer’s instructions. The CDC protocol was optimized for RT-dPCR: details are provided in Supporting information Table S1. Each reaction was prepared as a 20 µL volume consisting of 1× reaction mix, pre-mixed N1 primers/probe solution (1000 nM forward primer, 1000 nM reverse primer, 250 nM probe), and template (4 µL of nucleic acid extract). Each RT-dPCR experiment included positive and no-template controls (nuclease-free water).

Standard curve and control samples (described above) were reverse-transcribed, amplified, detected, and quantified by RT-dPCR. Dot plots showing examples of positive controls, test samples, and negative controls are provided in Fig. S1.

## Results and discussion

### Wastewater based epidemiology for detection of SARS-CoV-2

**Figure 1:**
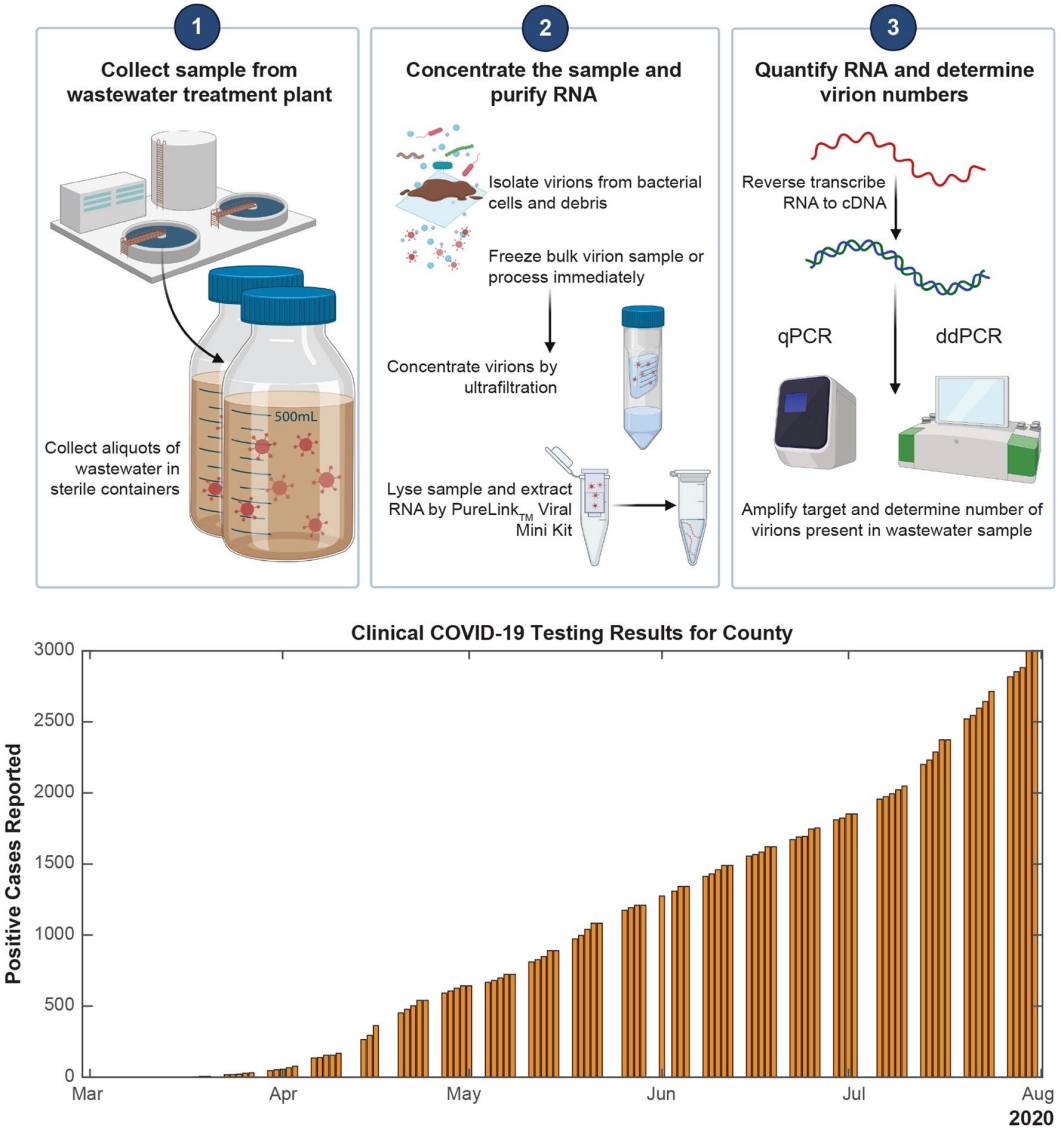
Wastewater-based monitoring for community-level COVID-19 tracking. Top: Workflow for sample collection through detection and quantification of SARS-CoV-2 using both quantitative and droplet digital PCR platforms (created with BioRender.com). Bottom: County health department data for positive cases reported by clinical testing within the county that encompasses the wastewater treatment plant (sampling site) in the date range relevant to sample collection^30^.

The first confirmed case in St. Joseph County was reported on March 12, 2020. Between that time and June 14, 2020, clinical COVID-19 testing was reserved for individuals experiencing two or more symptoms or a significant and rapid decline in health. Clinical COVID-19 testing for all residents of the state of Indiana became available on June 15, 2020. The cumulative number of cases reported in the county are shown in the bottom panel of Fig. 1. Since many cases of the disease are asymptomatic, the prevalence of the disease in the community was likely higher than the values shown.

Raw wastewater provides an opportunity to assess community-wide viral load and holds the potential to estimate disease occurrence in a catchment area^29^. We began wastewater sampling and processing for community-level COVID-19 tracking on March 12, 2020, which coincided with the first positive clinical test in the region (Fig. 1). Wastewater was sampled 53 times during the course of this 19-week study (March 12, 2020–July 24, 2020) from the wastewater treatment plant that serves ~ 21% (56,227/271,826) of the county’s population. We continued sampling and processing through July 24, 2020. During this time, the total number of clinically confirmed cases within the county grew to 2714, with a seven-day average of 46 cases per day^30^.

Samples were collected, transported, and processed according to the workflow depicted in Fig. 1, top panel. Samples were acquired for evaluation of the underlying bioanalytical methods and were not used to guide public health actions.

### Method development

Samples were collected, transported, and processed as noted in Fig. 1. Samples were split for analysis. Viral RNA was extracted from concentrated wastewater samples, reverse transcribed, amplified, and quantified using the CDC N1 primers for both qPCR and dPCR. The N1 copy number in each sample was measured in triplicate PCR reactions and adjusted to copy number per liter of wastewater.

It is important to note that SARS-CoV-2 RNA was detected in raw wastewater by both RT-qPCR and RT-dPCR on the same date as the first clinical case in the community. The cumulative number of positive cases in the county increased to 3000 by the date of the last sample in late July 2020.

**Figure 2:**
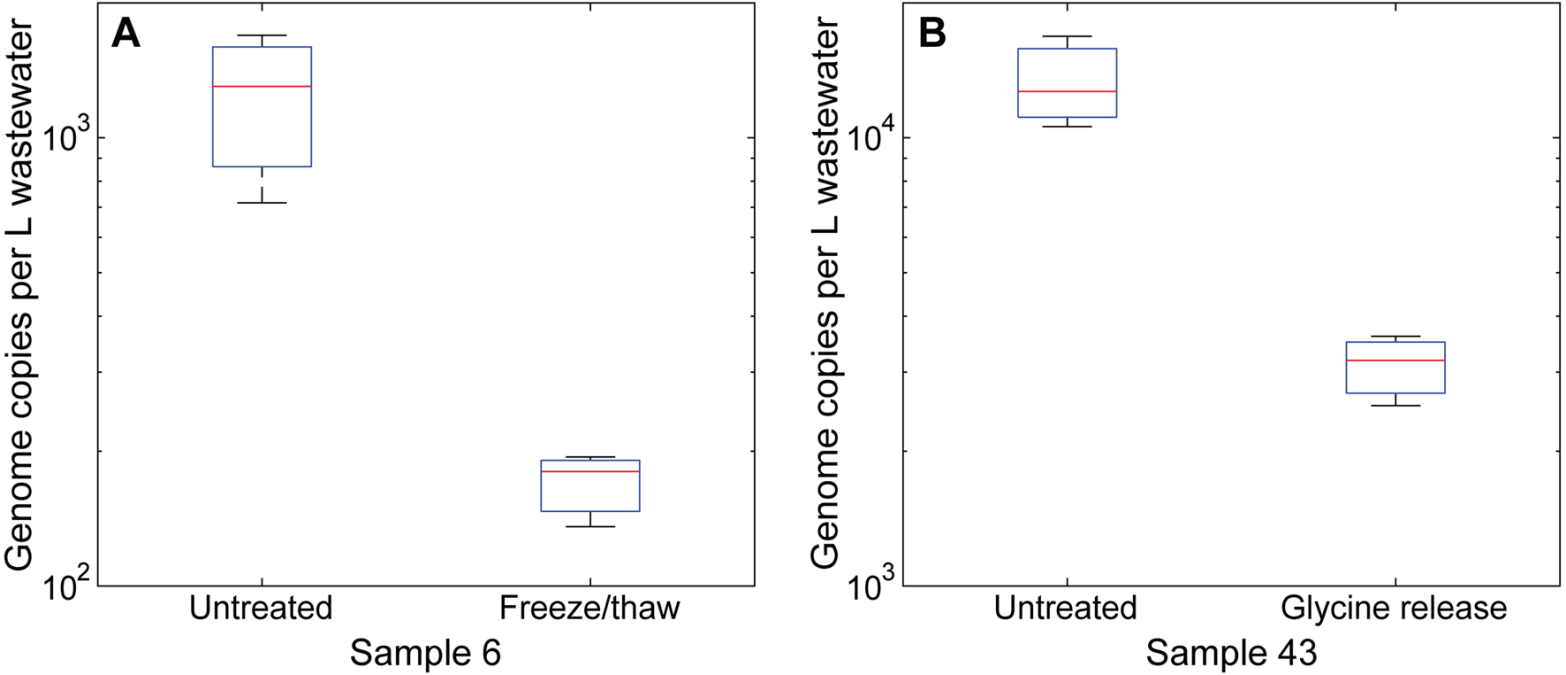
Effect of bulk sample processing on virion isolation from matched wastewater samples. Data were generated by RT-qPCR in technical triplicate. Box plots display minimum, maximum, interquartile range, and median.

We compared several protocols during this period, primarily due to the evolution in CDC recommendations (Fig. S2, Tables S2–S3). Three comparisons are of note. First, a subset of samples was frozen before analysis. To measure the effect of freeze/thaw cycles, we compared the N1 copy number in two independently processed samples. 500 mL of wastewater was reserved during the initial collection and processing of sample 6; that additional raw sample was stored at − 80 °C, thawed after 92 days in storage, and then processed. Figure 2 compares the results of the frozen bulk aliquot with the aliquot that was processed immediately after collection (Fig. 2A). There was a 7.2-fold decrease in genome copies detected, which demonstrates degradation of SARS-CoV-2 when stored in bulk and subjected to freeze/thaw prior to virion concentration.

Additional sample details are listed in Table S3, where storage is reported in days elapsed for each state: bulk, concentrate, RNA. Sample 6 ‘untreated’ was stored as concentrated virions for 60 days prior to extraction and purification of nucleic acids for analysis.

Second, we investigated the effect of a glycine release buffer. Sample 43 was split into two equal aliquots, which were processed in parallel immediately after collection; one sample was untreated and the other was treated with glycine release buffer (Fig. 2B). Comparison of these data show a 4.0-fold decrease in genome copies detected for treated samples, which suggest treatment of raw wastewater with glycine release buffer inhibits detection of SARS-CoV-2.

Third, we investigated the compound effect of compound treatments (both freezing and addition of glycine release buffer). Most samples subjected to compound treatment did not generate a positive signal. RT-dPCR outperformed RT-qPCR for these samples; RT-dPCR produced four positives to one positive by RT-qPCR in this period. These data suggest RT-dPCR is better suited if samples are preserved by freezing and are known to contain PCR inhibitors.

The effects of a single freeze–thaw cycle on a sample of bulk are shown in Fig. 2A. Comparison of the storage conditions and timeline for these samples shows increased stability of virions after concentration than virions in bulk liquid (i.e., direct from sampling site), frozen prior to analysis (Supporting information Table S3). Holohan et al*.* described a similar phenomenon, where rapid sample degradation was observed after a single freeze–thaw cycle, with respect to different compositions in transport media^31^.

Based on our apparent viral yields, we hypothesized that glycine release was negatively impacting recovery. Evidence for this can be seen in Fig. 2B. Glycine treatment to bulk material has been shown to enhance virion recovery from biosolids. However, from these data we discovered that at least in methods employing ultrafiltration/concentration the effect of glycine addition had severe effect on recovery. Addition of these reagents consequently reduced virion recovery, which is likely due to incompatibility with filtration devices potentially causing membrane clogging or blocking in concentration and in extraction^32,33^. In a recent review, Lu et al*.,* refer to glycine release treatment as a disadvantage, specifically referring to glycine and beef extract as “blocking agents” and warning that backflushing may be needed^34^.

Since freeze–thaw and glycine act on different stages in sample collection, we hypothesized that the effects of these ‘blocking agents’ would compound. Figure S2 supports the likelihood that the impact of these effects is additive. Reduction in viral loads detected to the extent shown here could render wastewater samples useless in surveillance of pathogens, with the most significant impact at early onset. Therefore, we recommend processing wastewater samples, without treatment, to at least a concentrated sample of virions prior to cryopreservation for best outcome.

**Figure 3:**
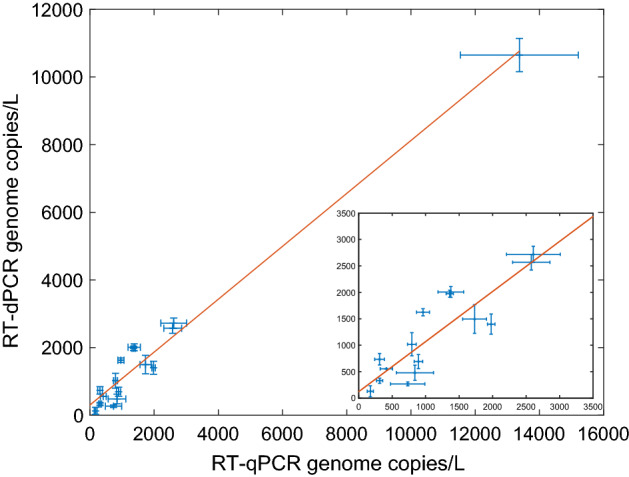
Comparison of RT-qPCR and RT-dPCR quantitative results for 16 paired, untreated samples. (Data presented ± standard deviation of the mean, unweighted least-squares fit shown in the orange line, r^2^ = 0.98, slope = 1.2. Insert—data and unweighted fit for lower amplitude signals, r = 0.87, slope = 0.82).

Finally, we compared the results from RT-qPCR with RT-dPCR for 16 fresh, untreated samples taken over the study shown in Fig. 3. The estimated viral loads from the two quantitative methods are highly correlated (r = 0.98, slope = 1.1). The relative precision in the measurements were similar ($\overline{\mathrm{x} }$/s = 0.12 for RT-dPCR, 0.15 for RT-qPCR), and the standard deviations of the mean from the two methods were uncorrelated (r = 0.26).

The variations in the replicates are due to both fundamental and experimental noise. Shot-noise in the sample compositions is a fundamental source of variation in signal that is important when dealing with small numbers of objects taken for analysis^35^. Experimental noise has a number of sources. In particular, viruses diluted by non-sewage sources of wastewater, such as rainwater entering the system. In addition, the variation in the wastewater matrix composition will introduce variation in PCR signal, because wastewater inevitably contains PCR inhibitors, whose concentration can vary from day to day. Clinical samples are less susceptible to these variations, because they typically contain higher viral titers and much more consistent sample matrix. We obtained water quality measurements from the wastewater plant including precipitation for the sampling days and attempted to correlate these variables with detected viral load, supporting information Table S4. No correlation was readily observed from the data available, likely reflecting numerous confounding factors, experimental noise in collection time, and chemical and biological properties of the wastewater inflow. We did observe a slight negative trend when comparing suspended solid levels and carbonaceous biochemical oxygen levels (CBOD5). In general, we observed lower levels of virions at values > 150 mg/L for dissolved organic matter.

### Comparison of measurement: controls

**Figure 4:**
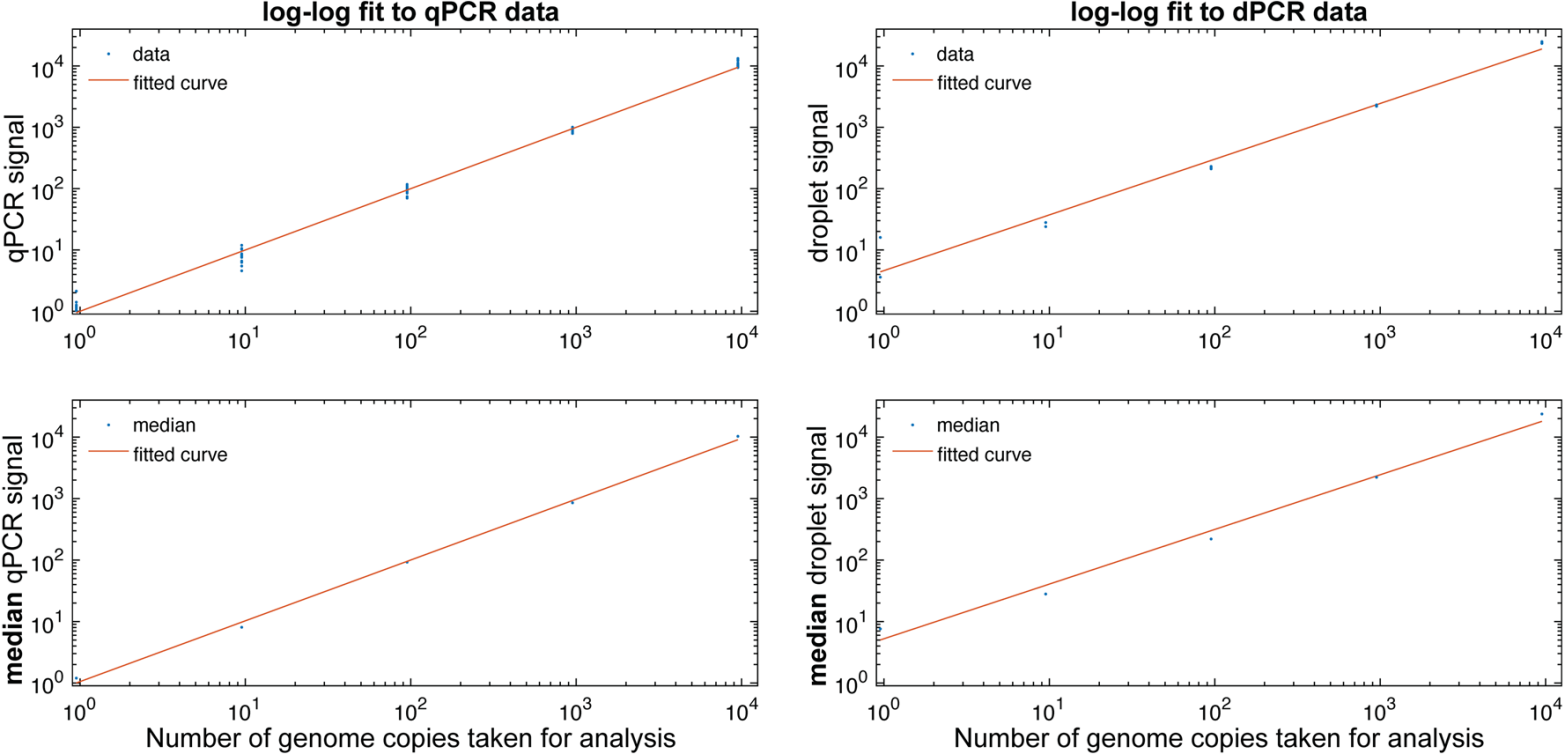
Quantitation of SARS-CoV-2 RNA N1 by RT-qPCR and RT-dPCR. Data were generated from technical replicates along a 1:10 dilution series of genomic RNA positive control material. Replicate data (top panels) and the replicate mean (bottom panels) along the dilution series are shown with a weighted fit to a straight line. The equations for the log–log RT-qPCR data: y = 1.0x − 5.3E−05 (r^2^ = 1.0), fitted RT-qPCR median: y = 1.0x + 2.3E−02 (r^2^ = 1.0), fitted RT-dPCR data: y = 0.9x + 0.7 (r^2^ = 1.0), fitted RT-dPCR median: y = 0.9x + 0.7 (r^2^ = 1.0).

Quantification of genomic RNA from SARS-CoV-2 was performed on both qPCR and dPCR instruments using the N1 assay, supporting information Table S5. Data were generated from technical replicates along a 1:10 dilution series of positive control material. Figure 4 shows replicate data (top panels) and the replicate mean (bottom panels) along the dilution series with a weighted fit to a straight line. The fit was used to estimate the N1 95% limit of detection (LOD) of copies per reaction: RT-qPCR produced LOD of 1.0 copy (95% CI 0.9–1.1), RT-qPCR produced median LOD of 1.1 copies (95% CI 0.7–1.6), RT-dPCR produced LOD of 5 copies (95% CI 2.8–7.5), RT-dPCR produced median LOD of 5 copies (95% CI 2.0–14).

Quantitation of genomic RNA extracted from heat-inactivated SARS-CoV-2 virions was performed on both RT-qPCR and RT-dPCR instruments using the N1 assay. Data were generated from technical replicates of extracted material that nominally contained 469 virions. The methods produced 83% (RT-qPCR) and 76% (RT-dPCR) recovery, using the calibration curve shown in Fig. 4. Applying these results, LOD for extracted material is calculated at 1.3 (RT-qPCR) and 6.5 (RT-dPCR) copies per reaction.

Using preprocessed RT-qPCR samples, LOD was estimated at ~ 3 genome copies per reaction for RT-qPCR (2.8 genome copies). These data are in agreement with RT-dPCR results from LOD experiments and calculations that were performed and published earlier (3.3 genome copies)^21,36,37^.

The applicability of either method often depends on sample conditions. It is frequently hypothesized that RT-dPCR is more tolerant of PCR inhibitors naturally present in biological samples, making it an obvious choice for the detection of SARS-CoV-2 RNA in wastewater samples^24,25^. However, recent studies show that isn’t necessarily the case. D’Aoust et al*.* compared RT-qPCR and RT-dPCR detection of SARS-CoV-2 RNA in wastewater influent solids and demonstrate dPCR experiences significant signal suppression compared to qPCR^33^. A similar trend is observed by Boogaerts et al*.* for the detection of SARS-CoV-2 RNA from influent wastewater (IWW), with mostly comparable results between the two methods^38^. However, in patient samples RT-dPCR is shown to provide fewer false-negatives than RT-qPCR, proving better for clinical detection of low-viral samples^39^.

## Conclusion

Wastewater is an attractive sample in surveillance for longitudinally testing for pathogens, in this case SARS-CoV-2. Wastewater analysis generates community-wide data to inform public health and reduce the costs associated with ‘between the waves’ monitoring and during quiescent periods. Here, we demonstrate that retrospective analysis of frozen samples is possible, although at the expense of sensitivity due to reduced recovery. Variability due to sample preparation methods was substantially larger than variability introduced from the two detection methods, qPCR and dPCR. Both approaches are comparable in sensitivity and generally agree on precision and accuracy. Matrix effects due to inhibition in the preparation of samples were not observed here, as the terminal detection of dPCR is generally less sensitive to these effects. We also observe that common accepted methods for preserving and increasing the release of virions from organic matrix were largely ineffective, and in some cases detrimental to simple cryoprotection, and freeze–thaw. As expected, processing samples immediately after collection or processing to a concentrated state prior to cryoprotection had the highest recovery and sensitivity, but retrospective analysis and longitudinal studies on stored samples adequately preserved the ability to make informed simple stratification of the samples (e.g. None detected, Few, Abundant). Finally, we only considered PCR-based assays. It would be interesting to include alternative detection technologies, including CRISPR/Cas12a based assays, or protein-detection in future comparisons^40,41^.

## Supplementary Information

Supplementary Information 1.

Supplementary Information 2.

## Data Availability

Data are presented in supporting information Tables S2–S5 of this manuscript.
