# Supplementary material for: Comparison of RT-dPCR and RT-qPCR and the effects of freeze–thaw cycle and glycine release buffer for wastewater SARS-CoV-2 analysis
Source: Sci Rep. 2022 Nov 30;12:20641. doi: 10.1038/s41598-022-25187-1 (PMC9709738; doi:10.1038/s41598-022-25187-1)
Supplement: Supplementary file 1 — Supplementary Information 1. [file 41598_2022_25187_MOESM1_ESM.docx]

**Supporting information**

**Comparison of RT-dPCR and RT-qPCR and the effects of freeze-thaw cycle and glycine release buffer for wastewater SARS-CoV-2 analysis**

Bonnie Jaskowski Huge ^1^, Devin North ^2^, C. Bruce Mousseau ^1^, Kyle Bibby ^2,3^, Norman J. Dovichi ^1,3^, Matthew M. Champion ^1,3*^

^1^Department of Chemistry and Biochemistry, ^2^Department of Civil and Environmental Engineering and Earth Sciences, and ^3^Berthiaume Institute for Precision Health,
University of Notre Dame, Notre Dame, IN 46556 USA

Corresponding author’s email - Matthew.M.Champion.8@nd.edu

**Contents**

**Figures**

**Figure S1 –** RT-dPCR dot plots showing examples of positive controls, test samples, and negative controls – Pg S3

**Figure S2 –** Comparison of qPCR (upper) and dPCR (lower) analysis of SARS-CoV-2 on matched wastewater and nucleic acid samples – Pg S4

**Tables**

**Table S1 –** Protocol details – Pg S5

**Table S2 –** Quantitative results for all replicates (samples) (see spreadsheet Tables S2-S5)

**Table S3 –** Sample collection, processing, and storage details (see spreadsheet Tables S2-S5)

**Table S4 –** Inflow data for wastewater facility (see spreadsheet Tables S2-S5)
**Table S5 –** Quantitative results for all replicates (controls) (see spreadsheet Tables S2-S5)

**Figure S1**

**
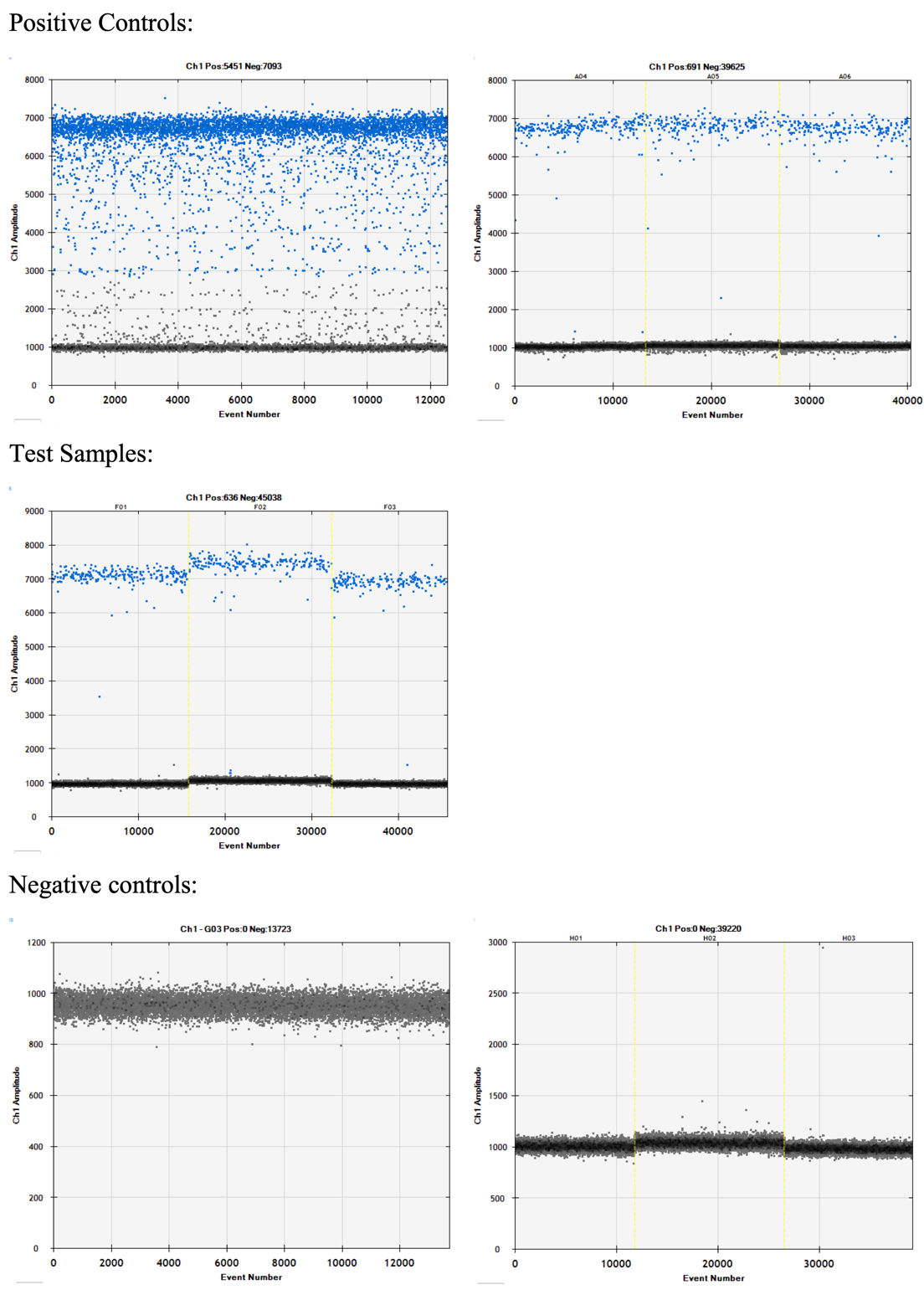
**

**Figure S1**. RT-dPCR dot plots showing examples of positive controls, test samples, and negative controls. Average droplet numbers are 14354.25, with a minimum of 10771 and a maximum of 17011. Droplets were generated manually using the QX200 Droplet Generator, and thresholding was done manually using negative controls to estimate RNA copy number. Average partition volume previously reported for BioRad ddPCR is 0.837 nL/droplet. Software Version 1.7.4.0917.

**Figure S2**

**Figure S2**. Comparison of qPCR (upper) and dPCR (lower) detection of SARS-CoV-2 on matched wastewater and nucleic acid samples. Data were normalized by template volume (2 µL per qPCR reaction and 4 µL per dPCR reaction), adjusted to genome copies per liter of wastewater, and plotted on log scale. Data were generated in triplicate for each sample and are color-coded by replicate. qPCR data are shown in the upper panel as a set of blue, red, and yellow bars per sample. dPCR data are shown in the lower panel as a set of light blue, purple, and green bars per sample. Symbols along the upper-most x-axis indicate sample treatment prior to virion concentration and nucleic acid extraction; glycine release (square) and freeze/thaw (circle).

**Table S1**. Protocol details:

| **Detector** | **Primers/probe reaction conc.** | **Template vol.** | **Reaction vol.** | **Thermal Cycling** |
| --- | --- | --- | --- | --- |
| RT-qPCR | Forward: 500 nM  Reverse: 500 nM  Probe: 125 nM | 2 µL | 20 µL | 25°C 2 min; 50°C 15 min; 95°C 2 min;  45 cycles: 95°C 3 s, 55°C 30 s ^1^ |
| RT-dPCR | Forward: 1000 nM  Reverse: 1000 nM  Probe: 250 nM | 4 µL | 20 µL | 50°C 60 min; 95°C 10 min;  40 cycles: 95°C 30 s, 59°C 60 s;  98°C 10 min ^2^ |

Primers/probe sequences:

Forward: 5’-GAC CCC AAA ATC AGC GAA AT-3’

Reverse: 5’-TCT GGT TAC TGC CAG TTG AAT CTG-3’

Probe: 5’-FAM-ACC CCG CAT TAC GTT TGG TGG ACC-BHQ1-3’

^1^CDC recommended protocol

^2^Protocol converted and optimized for RT-dPCR
